# A systematic review with procedural assessments and meta-analysis of Low Level Laser Therapy in lateral elbow tendinopathy (tennis elbow)

**DOI:** 10.1186/1471-2474-9-75

**Published:** 2008-05-29

**Authors:** Jan M Bjordal, Rodrigo AB Lopes-Martins, Jon Joensen, Christian Couppe, Anne E Ljunggren, Apostolos Stergioulas, Mark I Johnson

**Affiliations:** 1 Institute of Physiotherapy, Faculty of Health and Social Sciences, Bergen University College, Moellendalsvn. 6, 5009 Bergen, Norway; 2 Department of Public Health and Primary Health Care, Section of Physiotherapy Science University of Bergen, Kalfarveien 31, 5018 Bergen, Norway; 3 Department of Pharmacology, Institute of Biomedical Sciences, University of São Paulo. Av. Prof. Lineu Prestes, 1524, Butantan, 05508-900São Paulo – SP, Brazil; 4 University of Copenhagen – Institute of Sportsmedicine, Bispebjerg Hospital, Bispebjerg Bakke 23, 2400 Copenhagen NV, Denmark; 5 Faculty Human Movement & Quality Life, University of Peloponnese, 23100 Sparta, Greece; 6 Faculty of Health, Centre for Pain Research, Leeds Metropolitan University, Leeds, LS2 8AJ, UK

## Abstract

**Background:**

Recent reviews have indicated that low level level laser therapy (LLLT) is ineffective in lateral elbow tendinopathy (LET) without assessing validity of treatment procedures and doses or the influence of prior steroid injections.

**Methods:**

Systematic review with meta-analysis, with primary outcome measures of pain relief and/or global improvement and subgroup analyses of methodological quality, wavelengths and treatment procedures.

**Results:**

18 randomised placebo-controlled trials (RCTs) were identified with 13 RCTs (730 patients) meeting the criteria for meta-analysis. 12 RCTs satisfied half or more of the methodological criteria. Publication bias was detected by Egger's graphical test, which showed a negative direction of bias. Ten of the trials included patients with poor prognosis caused by failed steroid injections or other treatment failures, or long symptom duration or severe baseline pain. The weighted mean difference (WMD) for pain relief was 10.2 mm [95% CI: 3.0 to 17.5] and the RR for global improvement was 1.36 [1.16 to 1.60]. Trials which targeted acupuncture points reported negative results, as did trials with wavelengths 820, 830 and 1064 nm. In a subgroup of five trials with 904 nm lasers and one trial with 632 nm wavelength where the lateral elbow tendon insertions were directly irradiated, WMD for pain relief was 17.2 mm [95% CI: 8.5 to 25.9] and 14.0 mm [95% CI: 7.4 to 20.6] respectively, while RR for global pain improvement was only reported for 904 nm at 1.53 [95% CI: 1.28 to 1.83]. LLLT doses in this subgroup ranged between 0.5 and 7.2 Joules. Secondary outcome measures of painfree grip strength, pain pressure threshold, sick leave and follow-up data from 3 to 8 weeks after the end of treatment, showed consistently significant results in favour of the same LLLT subgroup (p < 0.02). No serious side-effects were reported.

**Conclusion:**

LLLT administered with optimal doses of 904 nm and possibly 632 nm wavelengths directly to the lateral elbow tendon insertions, seem to offer short-term pain relief and less disability in LET, both alone and in conjunction with an exercise regimen. This finding contradicts the conclusions of previous reviews which failed to assess treatment procedures, wavelengths and optimal doses.

## Background

Lateral elbow tendinopathy (LET) or "tennis elbow" is a common disorder with a prevalence of at least 1.7% [1], and occuring most often between the third and sixth decades of life. Physical strain may play a part in the development of LET, as the dominant arm is significantly more often affected than the non-dominant arm. The condition is largely self-limiting, and symptoms seem to resolve between 6 and 24 months in most patients [2].

A number of interventions have been suggested for LET. Steroid injections, non-steroidal anti-inflammatory drugs or a regimen of physiotherapy with various modalities, seem to be the most commonly applied treatments [3]. However, treatment effect sizes seem to be rather small, and recommendations have varied over the years. In several systematic reviews over the last decade [4,5], glucocorticoid steroid injections have been deemed effective, at least in the short-term. But in later well-designed trials evidence is found that intermediate and long-term effects of steroid injections groups yield consistently and significantly poorer outcomes than placebo injection groups, and physiotherapy or wait-and-see groups [6,7]. Nevertheless, steroid injections have been considered as the most thoroughly investigated intervention, with 13 randomized controlled trials comparing steroid injections to either placebo/local anaesthetic or another type of intervention [5]. Non-steroidal anti-inflammatory drugs (NSAIDs) have been found to achieve smaller short-term effect sizes than steroid injections [8], and topical application seems to be the best medication administration route [8] For oral administration of NSAIDs for LET, evidence is inconclusive from two heterogeneous trials only [9]. The positive short-term results of anti-inflammatory therapies in LET appear to partly contradict the recent paradigm in tendinopathy research, where LET is thought to be mainly a degenerative disorder with minimal inflammation [10,11].

Exercise therapy and stretching exercises have been used either alone or in conjunction with manipulation techniques or physical interventions. Although the sparse evidence makes it difficult to assess the separate effect of active exercises or stretching [12], four studies have found that either exercises alone [13], or in conjunction with a physiotherapy package, are more effective than placebo ultrasound therapy or wait-and-see controls. Also exercise therapy, particularly eccentric exercises, have been found effective in the intermediate term in tendinopathies of the Achilles, patellar or shoulder tendons [14-17]. There is some evidence suggesting that joint manipulation or mobilisation techniques either of the wrist, elbow or cervical spine may contribute to short-term effects in LET [18-20].

Among the physical interventions, ultrasound therapy has been considered to offer a small benefit over placebo from two small trials [12], but a well-designed and more recent trial did not find significant effects of ultrasound therapy in LET [21]. Reviewers have arrived at different conclusions for the effect of acupuncture [22,23]. In reviews of physical interventions for LET, conclusions may vary between reviews because of differences in the treatment procedures. A good example of this is the negative conclusion of the LET review for extracorporeal shockwave therapy (ESWT) by Buchbinder et al. [24], where a later review with in-depth assessments of treatment intervention protocols [25], found that a subgroup of trials with proper treatment procedures and adequate timing of outcomes gave a positive result.

Low level laser therapy (LLLT) has been available for nearly three decades, and scattered positive results have been countered by numerous negative trial results. Several systematic reviews have found no significant effects from LLLT, in musculoskeletal disorders in general [26], and in LET in particular [12,23,27]. In this perspective it may seem futile to perform yet another systematic review in this area. But none of these reviews evaluated the results separately for the different LLLT treatment procedures, laser wavelengths or doses involved. Neither did they implement evidence of the newly discovered biomodulatory mechanisms which are involved when LLLT is applied. During the last 5–6 years the annual number of published LLLT reports in Medline has increased from 25 to around 200. We recently made a review of this literature, and concluded that LLLT has an anti-inflammatory effect in 21 out of 24 controlled laboratory trials, and a biostimulatory effect on collagen production in 31 out of 36 trials [28]. Both of these effects were dose-dependent and could be induced by all wavelengths between 630 and 1064 nm with slight variations in therapeutic dose-ranges according to the wavelength used. The anti-inflammatory effect was seen in higher therapeutic dose-ranges than the biomodulatory effect on fibroblast cells and collagen fibre production. Diagnostic ultrasonography of tendinopathies has revealed that partial ruptures and tendon matrix degeneration are underdiagnosed if only physical examinations are made. Consequently, the stimulatory LLLT-effect on collagen fibre production should probably be beneficial for tendon repair. Another interesting feature was that LLLT with too high power densities or doses (above 100 mW/cm^2^), seemed to inhibit fibroblast activity [29] and collagen fibre production [30]. Six years ago we showed in a systematic review of tendinopathy, that the effect of LLLT is dose-dependent [31]. At the time, the accompanying editoral suggested that the advanced review design could become the new standard for reviewing empirical therapies with unknown optimal doses and procedural differences [32]. Steroids induce a down-regulation of cortisol receptors, and we recently discovered that the cortisol antagonist mifepristone completely diminished the anti-inflammatory effect of LLLT [33]. All these recent findings from the LLLT literature, prompted the World Association for Laser Therapy (WALT) to publish dosage recommendations and standards for the conductance of systematic reviews and meta-analyses last year [34]. One of the issues that has lacked attention is the validity of LLLT-application procedures in tendinopathy. To our knowledge there are only three valid irradiation techniques for LLLT in tendinopathies: a) direct irradiation of the tendon, b) irradiation of trigger points and c) irradiation of acupuncture points.

In this perspective and as our previous tendinopathy review [31] is becoming outdated, there seems to be a need for a new in-depth review of the effects of LLLT in LET where possible confounders are analyzed and subgroup analyses are performed.

## Methods

### Literature search

A literature search was performed on Medline, Embase, Cinahl, PedRo and the Cochrane Controlled Trial Register as advised by Dickersin et al. [35] for randomised controlled clinical trials. Key words were: Low level laser therapy OR low intensity laser therapy OR low energy laser therapy OR phototherapy OR HeNe laser OR IR laser OR GaAlAs OR GaAs OR diode laser OR NdYag, AND tendonitis OR lateral epicondylitis OR lateral epicondylopathy OR tennis elbow OR elbow tendonitis OR lateral epicondylalgia OR extensor carpi radialis tendonitis. Handsearching was also performed in national physiotherapy and medical journals from Norway, Denmark, Sweden, Holland, England, Canada and Australia. Additional information was gathered from researchers in the field.

### Inclusion criteria

The randomised controlled trials were subjected to the following seven inclusion criteria:

1) Diagnosis: Lateral elbow tendinopathy, operationalised as pain from the lateral elbow epicondyle upon finger or wrist extension

2) Treatment: LLLT with wavelengths in the range 632 – 1064 nm, irradiating either the tendon pathology, acupuncture points or trigger points

3) Design: Randomised parallel group design or crossover design

4) Blinding: Outcome assessors should be blinded

5) Control group: Placebo control groups or control groups receiving other non-laser interventions with at least 10 persons per group

6) Specific endpoints for pain intensity or global improvement of health measured within 1 – 52 weeks after inclusion.

### Outcome measures

#### Primary outcome measures

measured after the end of treatment, either as:

a) pain intensity on a 100 mm visual analogue scale (VAS) defined as the pooled estimate of the difference in change between the means of the treatment and the placebo control groups, weighted by the inverse of the pooled standard deviation of change for each study, i.e. weighted mean difference (WMD) of change between groups. The variance was calculated from the trial data and given as 95% confidence intervals [95% CI] in mm on VAS, or

b) improved global health status. This was defined as any one of the following categories: "improved", "good", "better", "much improved", "pain-free", "excellent". The numbers of "improved" patients were then pooled to calculate the relative risk for change in health status. A statistical software package (Revman 4.2) was used for calculations.

#### Secondary outcome measures

c) painfree grip strength (dynamometer, vigorimeter)

d) pain pressure threshold (algometer)

e) sick leave (days)

f) follow-up results at more than 1 week after the end of treatment for pain intensity (WMD) and/or improved global health status (RR) as described for the primary outcome measures

Due to possibility of measurement by different scales, the results for outcomes c) and d) are defined as the unitless pooled estimate of the difference in change between the mean of the treatment and the placebo control groups, weighted by the inverse of the pooled standard deviation of change for each study, i.e. standardised mean difference (SMD) of change between groups. The variance are calculated from the trial data and given as 95% confidence intervals.

### Analysis of bias, including methodological quality, funding source and patient selection

#### Positive bias direction, caused by flaws in trial methodology, funding source

Trials were subjected to methodological assessments by the 10 point Delphi/PedRo checklists [36]. as trials of weaker methodology have been found to exaggerate results in a positive direction [37]. As profit funding has been shown to affect trial conclusions in a positive direction [38], analysis of funding sources was also performed.

#### Negative bias direction, caused by poor prognosis or effective co-interventions

LET patients with long symptom duration and high baseline pain intensity are found to have significantly poorer prognosis in a trial with symptom durations of 8 to 21 weeks [2]. Recent steroid injections have been reported to negatively affect prognosis in LET over a period of 3–12 months after injections [6]. Patient selection of known responders only has been shown to inflate trial results with 38% [39], and consequently the inclusion of non-responders to treatments is likely to deflate effect sizes. Exercise therapy has been found effective in LET [13] and other tendinopathies [17], and the use of exercise therapy as a co-intervention may also deflate effect sizes or erase positive effects of LLLT. Consequently, we decided to analyze the included trials for presence of long symptom duration, treatment and treatment failures prior to inclusion, and effective co-interventions.

## Results

### Literature search results

**Figure 1:**
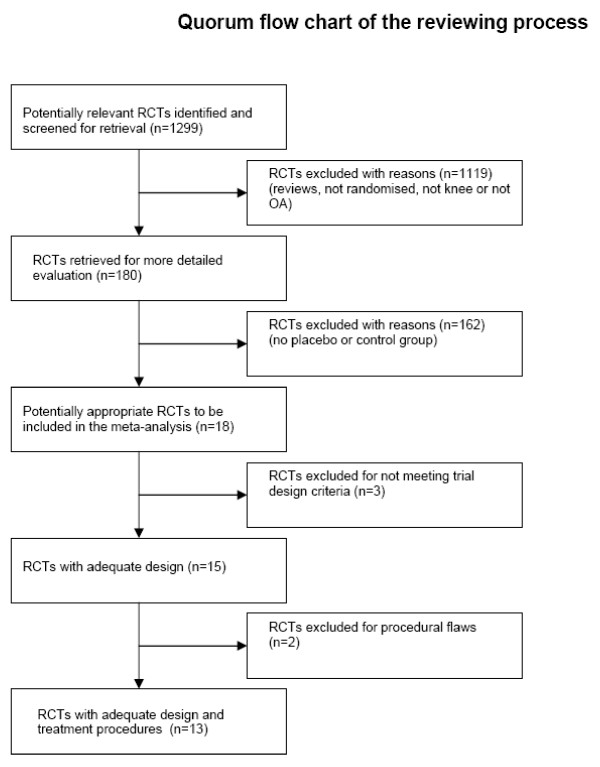
**Quorum flow chart. **Quorum flow chart of the steps in the reviewing process.

The literature search identified 1299 potentially relevant articles that were assessed by their abstracts. 1119 abstracts were excluded as irrelevant, 180 full trial reports were evaluated, and 18 trials met the inclusion criterion for randomisation (Figure 1).

However a further three randomised trials had to be excluded for not meeting the *a priori *trial design criteria for sample size in control group, specific endpoints or blinding. The results of this assessment are summarised in Table 1.

**Table 1:** Randomised LLLT-trials excluded for not meeting trial design criteria for diagnosis, blinding or specific endpoints.

**Study by first author**	**Year**	**Method score**	**Laser wavelength**	**Application technique**	**Result**	**Reason for exclusion**
Mulcahy [40]	1995	5	904	Not stated	No significant differences between active and placebo LLLT	Does not satisfy control group criterion: Lacks sufficient patient numbers in placebo control group as only 3 patients had tendinopathy
Simunovic [41]	1998	3	830	Tendon + Trigger Points	LLLT significantly better than placebo	Does not satisfy criterion for specific endpoint and standard number of treatments: Only bilateral conditions were given placebo treatment, but data for this group were not presented
Vasseljen [42]	1992	5	904	Tendon	Traditional physiotherapy significantly better than LLLT	Does not satisfy blinding criterion: Neither therapist, patients or observers were blinded in the traditional physiotherapy group

Trial characteristics by first author, method score, laser wavelength in nanometer, laser application technique, trial results and reason for exclusion.

### Analysis of treatment procedures

**Figure 2:**
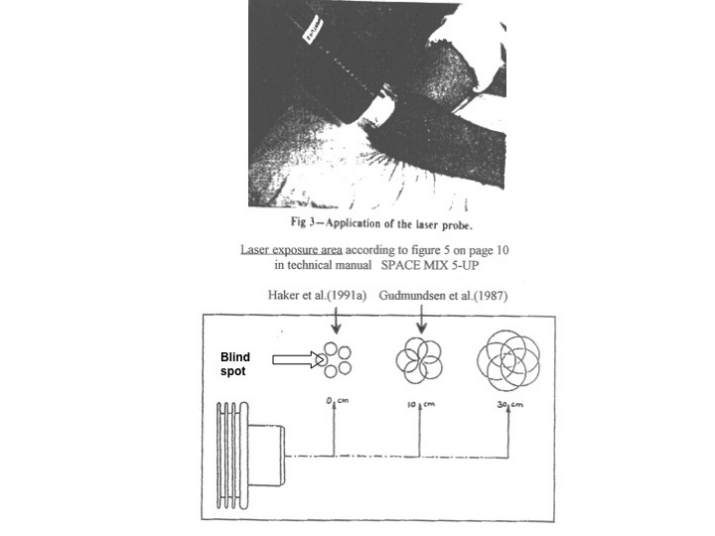
**Photograph showing laser therapy procedure with laser head in skin contact in trial by Haker et al.** The photograph is taken the trial report in from Archives of Physical Medicine 1991. The drawing of the laser spot sizes at different distances is taken from the manual of Space Mix 5 Mid-Laser (Space s.r.l, Italy).

The remaining 15 trials were then evaluated for adequacy of their treatment procedures for active laser and placebo laser for adherence to either of the three valid application techniques (inclusion criterion 2). This resulted in the exclusion of 2 trials (Table 2, Figure 2).

**Table 2:** Randomised LLLT-trials excluded for not meeting criteria of valid procedures for active laser and placebo laser treatment.

**Study by first author**	**Method score**	**Wave-length**	**Application technique**	**Result**	**Reason for exclusion**
Haker [43]	6	904	Tendon	No significant differences	Photograph in trial report shows that the laser probe was kept in skin contact and thereby violated the manufacturers' recommendation of a keeping the laser head at a distance of 10 cm. This violation caused a central blind spot of ca 3 cm^2 ^which left the tendon pathology unexposed to LLLT (See Figure 2)
Siebert [44]	6	904 + 632	Tendon	No significant differences	Active laser treatment to the placebo group received red 632 nm LLLT, which we calculated to be (2.25J), which again is an adequate LLLT dose. Consequently this trials lacks a placebo or non-laser control group

Trial characteristics given by first author, method score, laser wavelength, laser application technique, trial results and reason for exclusion.

### Publication bias

**Figure 3:**
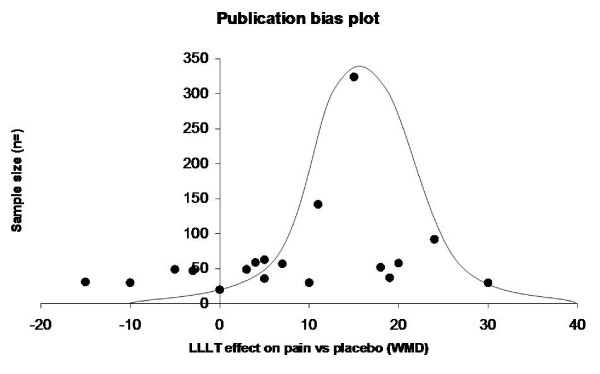
Funnel plot of published trial results given by WMD for pain relief over placebo measured on 100 mm VAS (x-axis), and sample size (y-axis).

The five excluded RCTs [40-44] were taken into the publication bias analysis by a graphical plot as advised by Egger [45]. Four [40-42,44] out of the five excluded trials with grave methodological and procedural flaws, were small and reported negative results. Three trials with negative results for LLLT were performed by the same research group [40,46,47] although this group also reported a positive outcome [50]. Three of these trials met the eligibility criteria for this review and were included in the meta-analysis [46,47,50]. The five largest trials [43,48-51] all presented positive results, although Simunovic et al. [43] was excluded from our meta-analyses for variable timing of endpoints as stated above. Significant asymmetry was noted in the funnel plot, indicating a considerable degree of negative publication bias (Figure 3).

### Bias analysis of 13 included trials

#### Positive bias detection – poor methodological quality and for-profit funding sources

The final study sample consisted of 730 patients in 13 trials. The mean and median methodological score was 6.5, and only one trial did not satisfy half or more methodological criteria [52]. Two trials used the acupoints application technique [46,47], while the remaining eleven trials used the tendon application technique. None of the trials stated funding from laser manufacturing companies or had authors with affiliations to laser manufacturers. The trial characteristics and the sum methodological scores are listed in Table 3.

**Table 3:** Included randomised LLLT-trials.

**Study by first author**	**Method score**	**Patient numbers**	**Application technique**	**Control**	**Trial results**
Basford [53]	8	47	Tendon	Placebo	0
Gudmundsen [51]	6	92	Tendon	Placebo	++
Haker [46]	7	49	Acupoints	Placebo	0
Haker [50]	6	58	Tendon	Placebo	+
Krashenninikoff [54]	6	36	Tendon	Placebo	0
Lam [55]	7	37	Tendon	Placebo	++
Løgdberg-Anderson [49]	7	142	Tendon	Placebo	++
Lundeberg [47]	6	57	Acupoints	Placebo	0
Oken [56]	7	59	Tendon	UL, Brace	++
Palmieri [57]	6	30	Tendon	Placebo	++
Papadoupolos [52]	4	31	Tendon	Placebo	-
Stergioulas [48]	7	62	Tendon	Placebo	++
Vasseljen [58]	8	30	Tendon	Placebo	+
Total	6.5(Mean)	730			

Trial characteristics by first author, method score, laser application technique, control group type, trial results. The abbreviations used are determined by the following categories: (-) means a result in favour of the control group, (0) means a non-significant result, (+) means a positive result for LLLT in at least one outcome measure, and (++) means a consistent positive results for more than one outcome measure.

#### Subgroup analysis for methodological quality

The pre-planned subgroup analysis by methodological quality was not performed as all but a single low quality trial were rated fairly similarly with 6–8 criteria fulfilled out of 10 possible criteria. Minor inter-observer differences have been reported for methodological scorings by the Pedro criteria list [36], and the variance could be within the range of measurement error for this methodological criteria list [53]. In addition, fulfilment of more than 50% of methodological criteria is often considered as a threshold for acceptable quality [54], and all but one trial with negative results were assessed with scores above this threshold. Consequently, we considered a separate subgroup analysis by methodological quality to be unnecessary to perform.

#### Negative bias detection – inclusion of patients with poor prognostic factors and effective co-interventions

Three trials reported details confirming enrolment of patients without poor prognosis [48,55,56]. In two of these trials [55,56], both active and placebo groups received concurrent exercise therapy, which may have deflated effect size. Seven trials reported demographic data affirmative on the inclusion of LET patients with poor prognosis, which are likely to deflate effect sizes. Results for possible confounding factors which may deflate effect sizes are summarized in Table S4, Additional file 1.

#### Assessment of LLLT procedures and treatment variables

There was considerable heterogeneity in the treatment procedures and LLLT doses used in the included trials. Treatment characteristics for the 11 trials which used direct irradiation of tendon pathology are listed in Table S5, Additional file 1.

Treatment characteristics for trials which used acupoint irradiation are listed in Table S6, Additional file 1.

### Outcomes and effect sizes

#### Dichotomized trial results

Eight out of thirteen trials (62%) reported one or more outcome measures in favour of LLLT over placebo. Eleven trials used the tendon application technique, and eight (73%) of these trials reported positive results for one or more outcome measures (Table 3). All seven trials using 904 nm wavelength and the tendon application technique yielded positive results [48-51,55-57], whereas three trials using lasers with 820/30 nm [58,52]and 1064 nm [59] wavelengths found no significant effect of LLLT. A single trial administering LLLT with a wavelength of 632 nm [60], also found significantly better results for the LLLT group. In the two trials where LLLT was administered to acupuncture points [46,47], no significant differences between LLLT and placebo were found for any of the outcome measures.

### Meta-analyses of effects

#### Primary outcomes

**Figure 4:**
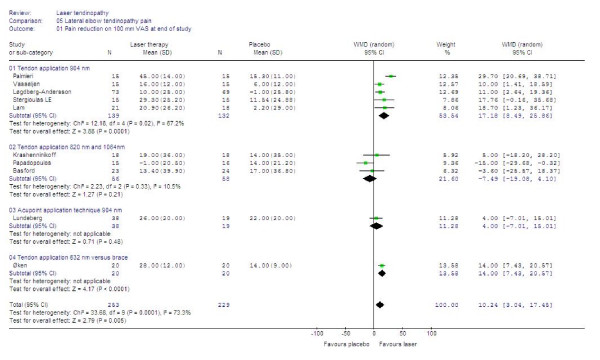
**End of treatment results for LLLT measured as the WMD pain reduction on 100 mm VAS. **Trials are subgrouped by application technique and wavelengths, and combined results are shown as total at the bottom of the table. Plots on the right hand side of the middle line indicate that the LLLT effect is superior to the control treatment.

Continuous data for pain relief was available from 10 trials in a way which made statistical pooling possible. At the first observation after the end of the treatment period, LLLT was significantly better than controls with a WMD of 10. 2 mm [95% CI: 3.0 to 17.5] in favour of LLLT on a 100 mm VAS (p = 0.005). In a subgroup of five trials [48,50,55-57] where 904 nm LLLT was administered directly to the tendon, LLLT reduced pain by 17.2 mm [95% CI: 8.5 to 25.9] more than placebo (p = 0.0001). One trial [60] with 632 nm LLLT, showed significantly better results for LLLT than a wrist brace and ultrasound therapy, but none of the results from trials with wavelengths of 820 nm or 1064 nm, or acupoint application technique were significantly different from placebo. The results are summarized in Figure 4.

**Figure 5:**
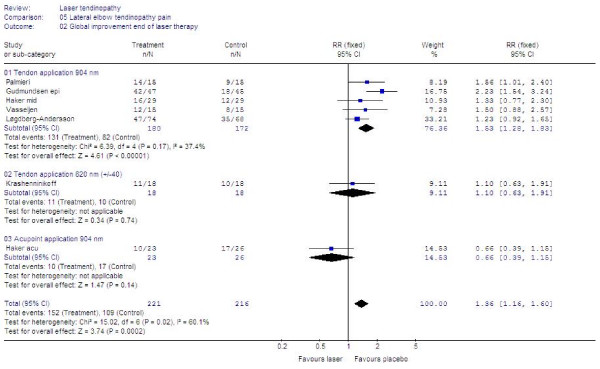
**End of treatment results for LLLT measured as global improvement. **Trials are subgrouped by application technique and wavelengths, and their combined results are shown as total at the bottom of the table. Plots on the right hand side of the middle line indicate that the LLLT effect is superior to the control treatment.

Seven trials [46,49-51,55,57,58] presented data in a way which allowed us to pool data for global improvement. LLLT was significantly better than placebo with an overall relative risk for improvement at 1.36 [95% CI: 1.16 to 1.60] (p = 0.002). In a subgroup of five trials [49-51,55,57] where 904 nm LLLT was used to irradiate the symptomatic tendon, the relative risk for global improvement was significantly better than placebo at 1.53 [95% CI 1.28 to 1.83] (p < 0.0001). In the remaining two trials [46,58] where LLLT was administered to acupoints or with 820 nm wavelength, the relative risk for global improvement was not significantly different from placebo at 0.80 [95% CI 0.50 to 1.22]. The results are summarized in Figure 5.

#### Secondary outcomes

**Figure 6:**
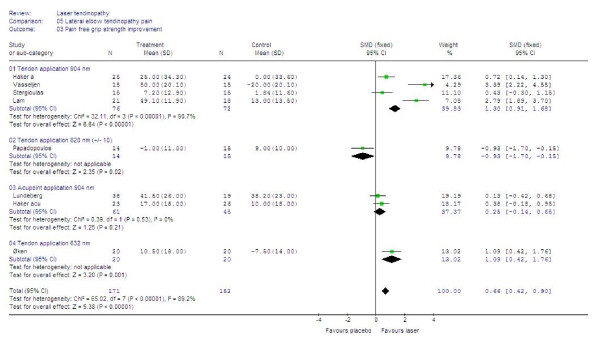
**End of treatment results for LLLT measured as the SMD for pain-free grip strength.** Trials are subgrouped by application technique and wavelengths, and their combined results are shown as total at the bottom of the table. Plots on the right hand side of the middle line indicate that the LLLT effect is superior to the control treatment.

Painfree grip strength showed significantly better results after LLLT than placebo with SMDs of 0.66 [95% CI: 0.42 to 0.90] [p < 0.0001). When trials were subgrouped by application technique and wavelengths, only trials with irradiation of tendons and wavelengths 632 nm [60] or 904 nm [48,49,56,57], showed positive results versus control with SMDs at 1.09 [95% CI: 0.42 to 1.76] and 1.30 [95% CI: 0.91 to 1.68], respectively. The results are summarized in Figure 6.

**Figure 7:**
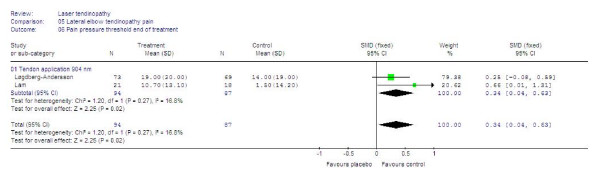
**End of treatment results for LLLT measured as the SMD for pain pressure threshold. **Only trials using the tendon application technique and 904 nm wavelength were available, and their combined results are shown as the total at the bottom of the table. Plots on the right hand side of the middle line indicate that the LLLT effect is superior to the control treatment.

Two trials with 904 nm wavelength using application technique with tendon irradiation [50,56] reported a small, but significantly elevated pain pressure threshold with SMD at 0.34 [95% CI: 0.04 to 0.63] (p = 0.02), The results are summarized in Figure 7.

#### Sick leave

One trial with 904 nm LLLT administered directly over the tendon insertion, presented sick leave data [51]. The relative risk for not being sicklisted after treatment was significantly in favour of LLLT at 2.25 [95% CI: 1.25 to 4.06] (p = 0.0005).

#### Follow-up

**Figure 8:**
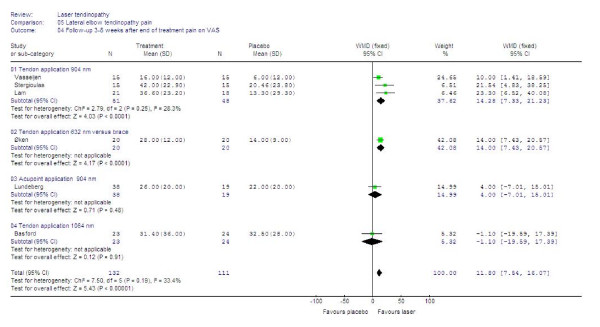
**Follow-up results at 3–8 weeks after end of treatment for LLLT measured as the WMD for pain reduction on 100 mm VAS.** Trials are subgrouped by application technique and wavelengths, and combined results are shown as total at the bottom of the table. Plots on the right hand side of the middle line indicate that the LLLT effect is superior to the control treatment.

**Figure 9:**
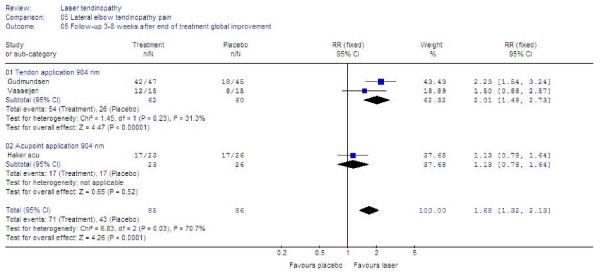
**Follow-up results at 3–8 weeks after the end of treatment measured as the relative risk for global improvement for LLLT compared to placebo. **Trials are subgrouped by application technique and wavelengths, and combined results are shown as total at the bottom of the table. Plots on the right hand side of the middle line indicate that the LLLT effect is superior to the control treatment.

Six of the trials provided continuous follow-up data on a 100 mm VAS measured between 3 and 8 weeks after the end of treatment [47,48,56,57,59,60]. The combined WMD was 11.30 mm [95% CI: 7.5 to 16.1] in favour of LLLT. For global improvement, three trials [46,51,57] provided data suitable for statistical pooling, and the RR was calculated to 1.68 [95% CI: 1.32 to 2.13] in favour of LLLT. Subgroup analyses showed that three trials [48,56,57] administering 904 nm LLLT directly over the tendon, WMD improved to 14.3 [95% CI: 7.3 to 21.3] and RR for improvement to 2.01 [95%CI: 1.48 to 2.73] in favour of LLLT, while a single trial [60] with 632 nm wavelength and the same application procedure reported WMD of 14.0 [95%CI: 7.0 to 20.6]. The results are summarized in Figures 8 and 9.

Only two trials using the tendon application technique with 904 nm wavelengths reported follow-up results beyond 8 weeks. They reported persisting significant improvement after LLLT for PFS at 3 months (SMD 0.40 [95%CI: 0.05 to 0.75]) [49], and significantly less patients with no or minor pain at work at 5.5 months (RR = 2.1 [95%CI: 1 to 4.3]) [57], respectively. Other outcomes were not significantly different beyond 8 weeks. For the two trials using acupoint irradiation [46,47], no significant differences were found at any of the follow-up sessions.

#### Side-effects and compliance

Treatment was generally well tolerated and no adverse events were reported. Compliance was high ranging from 100% to 91% in all but two trials [48,58]. One of these trials [48] had a considerably longer treatment period (8 weeks) than the other trials (median 3 weeks), and all withdrawals were caused by lack of effects. In another trial [58] using 830 nm wavelength, an exceptionally high withdrawal/dropout rate of 15% occurred after a single treatment session without any given reason.

## Discussion

In this review, we found that most RCTs of LLLT for LET were of acceptable methodological quality. This finding is in line with previous reviews [12,23,27], although there were some differences between reviewers in methodological scores for individual trials. RCTs of LLLT are of similar methodological quality and include similar sample sizes as RCTS included in recent reviews of corticosteroid injections [5] and topical or oral NSAIDs [8]. Two of the previous reviews of LLLT for LET found only six RCTs [12,23], whereas an earlier review found ten RCTs [27], and excluded one RCT for methodological shortcomings [43]. We used broader searching criteria in our review and had no language restrictions. This resulted in 18 potentially eligible RCTs. We excluded one RCT for not meeting the inclusion criteria of specific endpoints [43] and another two RCTs for complete lack of blinding [44] and a lack of an LET control group [42]. None of the previous LET reviews assessed the LLLT regimen for procedural errors, while our procedural assessments resulted in exclusion of another two RCTs with grave procedural errors, such as leaving the tendon insertion and acupoints unirradiated [40] and giving adequate LLLT to the placebo group [61]. These exclusions resulted in 13 RCTs being eligible for our review which is twice the number of RCTs included in two of the previously published reviews[12,23].

Previous LET-reviews of LLLT [12,23,27] and pharmacological interventions like NSAID [8] or corticosteroid injections [5] have not assessed possible bias from for-profit funding sources or publication bias. Our analysis revealed that bias from for-profit funding was largely absent in the available LLLT material and that trials were performed by independent research groups receiving funding from internal sources or non-profit organisations. This feature of the LLLT literature is definitely different from pharmacological pain treatments where up to 83% of trials may be industry-funded [62]. A second feature of the LLLT-literature is that publication bias seems to go in a negative direction. This is distinctly different from the drug trials [63,64] where positive results have been found to account for up to 85% of the published trials in single journals [63], although this bias seems to be lesser or absent in high impact journals [64]. Our review suggests that LLLT trials reporting negative results are more likely to be published than trials with positive results. To our knowledge we are the first to demonstrate such bias, but such negative publishing bias is probably not unique to LLLT, and it may also be present for other electrophysical agents including TENS and acupuncture. We were surprised to see how large well-designed positive trials of LLLT [51,50] were published in unlisted journals or journals with low-impact factor, and how small negative trials [46], often with grave methodological [42] or procedural flaws [40] were published in higher ranking journals. This may reflect a predominance of RCTs designed using drug-research methodology paradigms without due consideration given to adequacy of the technique used in delivering LLLT, leading to under dosing and negative outcome bias [65]. In addition, it has been that documented drug sponsorship of research activities may influence guideline panels, journal editors and referees [66,67] leading to negative views on non-drug treatments such as LLLT as reflected in editorials in pain journals [68] and national medical journals [69].

Despite these concerns, we believe that the positive overall results of this review need to interpreted with some caution. They arise from a subgroup of 7 out of the 13 included trials [48-51,55-57]. These 7 trials had a narrowly defined LLLT regimen where lasers of 904 nm wavelength with low output (5–50 mW) were used to irradiate the tendon insertion at the lateral elbow using 2–6 points or an area of 5 cm^2 ^and doses of 0.25–1.2 Joules per point/area. The positive results for this subgroup of trials were consistent across outcomes of pain and function, and significance persisted for at least 3–8 weeks after the end of treatment, in spite of several factors which may have deflated effect sizes.

For the red 632 nm wavelength which has a poorer skin penetration ability [70], a single trial [60] with a higher dose (6 Joules) seemed to be equally effective as the lower doses of 904 nm used in the seven positive trials. These LLLT-doses are well within the therapeutic windows for reducing inflammation, increasing fibroblast activity and collagen fibre synthesis, and the dosage recommendations suggested by WALT [71].

The negative results for the 830 nm GaAlAs and 1064 nm NdYag lasers can be attributed to several factors such as too high doses, too high power density or the inclusion of patients with poor prognosis from long symptom duration and prior steroid injections. These wavelengths have previously been found effective in some tendon animal studies and in other locations such as shoulder tendinopathies [72,73]. At this time it is not possible to draw firm conclusions about the clinical suitability of wavelengths 820, 830 and 1064 nm in LET treatment, but the lack of evidence of effects indicates that they cannot be recommended as LET treatment before new research findings have established their possible effectiveness. The lack of effect for these lasers may also serve as a reminder that higher doses is not always best. We have been witnessing a tendency where newly developed lasers with these wavelengths are being marketed with ever-increasing power and power densities. This may be inappropriate because current knowledge about LLLT mechanisms and dose-response patterns at higher powers is inconsistent or lacking.

The positive results for combining LLLT of 904 nm wavelength with an exercise regimen, are encouraging. We would have thought that exercise therapy could have erased possible positive effects of LLLT, but the results showed an added value in terms of a more rapid recovery when LLLT was used in conjunction with an exercise regimen. This may indicate that exercise therapy can be more effective when inflammation is kept under control. Adding LLLT to regimens with eccentric and stretching exercises reduced recovery time by 4 and 8 weeks in two trials [48,56]. For this reason, LLLT should be considered as an adjunct, not an alternative, to exercise therapy and stretching.

Based on the above findings, LLLT should be considered as an alternative therapy to commonly used pharmacological agents in LET management. Cochrane-based reviews of NSAIDs [8] and corticosteroid injections [5] have found evidence of short-term effects within 4 and 6 weeks, respectively. The short-term reduction in pain intensity after corticosteroid injections may appear to have a more rapid onset and may also be larger in effect size than after LLLT. But on the other hand, the available LLLT-material is confounded by factors capable of deflating effect sizes. In this perspective, there is a need for more high quality trials with head-to-head comparison of short-term effects between LLLT and corticosteroid injections. In the longer term, NSAIDs seems to be ineffective and corticosteroid injections seem to be harmful both at 26 and at 52 weeks [6]. For LLLT there are some significant long-term effects found at 8, 12 and 24 weeks after the end of treatment.

## Conclusion

The available material suggests that LLLT is safe and effective, and that LLLT acts in a dose-dependent manner by biological mechanisms which modulate both tendon inflammation and tendon repair processes. With the recent discovery that long-term prognosis is significantly worse for corticosteroid injections than placebo in LET, LLLT irradiation with 904 nm wavelength aimed at the tendon insertion at the lateral elbow is emerging as a safe and effective alternative to corticosteroid injections and NSAIDs. LLLT also seems to work well when added to exercise and stretching regimens. There is a need for future trials to compare adjunctive pain treatments such as LLLT with commonly used pharmacological agents.

## Competing interests

The authors declare that they have no competing interests.

## Authors' contributions

JMB had the original idea, which was developed through lengthy discussions with contributions from RABL-M, JJ, CC, AEL, AS and MIJ. The literature search, including handsearching, was performed by all members of the author team. The first draft was written by JMB, RABL-M and JJ, and revised by AS and MIJ. Methodological assessments of trials were performed by JMB, AEL, CC, AS. The statistical analysis was performed by JMB, RABL-M, JJ and MIJ. The final linguistic revision was performed by MIJ and all members of the author team read and commented on the manuscript before submission.

## Pre-publication history

The pre-publication history for this paper can be accessed here:



## Supplementary Material

Additional file 1 Tables 4-6. Click here for file
