# Supplementary material for: A systematic review with procedural assessments and meta-analysis of Low Level Laser Therapy in lateral elbow tendinopathy (tennis elbow)
Source: BMC Musculoskelet Disord. 2008 May 29;9:75. doi: 10.1186/1471-2474-9-75 (PMC2442599; doi:10.1186/1471-2474-9-75)
Supplement: Additional file 1 — Tables 4-6. [file 1471-2474-9-75-S1.doc]

Table 4

| **Study by first author** | **A)**  **Steroid injections**  **( > 20%)** | **B)**  **Mean symptom duration** | **C)**  **Mean baseline pain** | **D)**  **Prior treatment failures** | **E)**  **Concurrent exercise therapy** | **Deflated effect size due to patient selection bias** |
| --- | --- | --- | --- | --- | --- | --- |
| Basford [53] | 6/23 | 25 | 48 | ? | No | Yes (A, B) |
| Gudmundsen[51] | ? | 19 | ? | 134/92* | No | Possible (A,C,D)** |
| Haker [50] | 17/25 | 52 | ? | 20/25 | No | Yes (A,B,D) |
| Haker [46] | 18/49* | 26 | ? | 38/49* | No | Yes (A,B,D) |
| Krashenninikoff [54] | ? | 9 | 55 | ? | No | Possible (A, D) |
| Lam [55] | 0 | 13 | 51 | 0 | Eccentric + stretching | Possible (E) |
| Lundeberg [47] | 0 | > 12 | ? | ? | No | Possible (B,C,D) |
| Løgdberg-Andersson [49] | 8/73 | 26 | 36 | 54/73 | No | Yes (B)* * |
| Oken [56] | 0 (not < 6 weeks before) | 24 | 71 | ? | Strengthen-ing and stretching | Yes (B,C,E) |
| Palmieri [57] | 0 | 5 | 65 | 0 | No | No |
| Papadopoulos [52] | 11/14 | 28 | 45 | 21/14 | Yes (no details) | Yes (A,B,C,D) |
| Stergioulas [48] | 0 | 6 | 53 | 0 | Eccentric + stretching | No |
| Vasseljen [58] | 5/15 | 17 | 43 | 8/15 | No | Yes (A, E) |

Table 4. Trials given by first author, and details of affirmative negative bias caused by A) prior steroid injections >20% in LLLT group, B) mean symptom duration >21 weeks, C) baseline pain intensity > 60 mm on 100 mm VAS, and possible negative bias caused by D) selection of known non-responders to treatment or E) co-interventions with known effectiveness. Summary bias evaluation by “Yes”, if affirmative bias from A, B or C is found, and “Possible”, if data for A, B or C is not reported or if possible bias from selection of non-responders (D) or effective co-interventions. (*Only data for total trial sample reported, ** significantly better result (not specified) for subgroup with shorter symptom duration than for whole patient sample results)

Table 5

| **Study by first author** | **Laser wavelength (nm)** | **Laser mean output (mW)** | **Energy dose per point (J)** | **Total energy dose per session (J)** | **Power density (mW/cm^2^)** | **Points irradiated** | **Total irradiation time (s)** | **No. therapy sessions** |
| --- | --- | --- | --- | --- | --- | --- | --- | --- |
| Basford [53] | 1064 | 4080 | 12.2 | 12.2 | 204 | 7 | 60 | 12 |
| Gudmundsen[51] | 904 | 12 | 1.2 | 1.2 | 3 | 4.6 cm^2^ area | 480 | 8 |
| Haker [50] | 904 | 12 | 0.36 | 2.16 | 24 | 6 | 180 | 10 |
| Krashenninikoff [54] | 830 | 30 | 3.6 | 7.2* | 110 | ? | 120 | 8 (max.) |
| Lam [55] | 904 | 25 | 0.28 | 0.66 | 208 | 2.4 | 22 | 9 |
| Løgdberg-Andersson [49] | 904 | 8 | 0.25* | 0.5 | 24 | 2* | 62 | 6 |
| Oken [56] | 632 | 10 | 6 | 6 | ? | 1 | 600 | 10 |
| Palmieri [57] | 904 | 3 | 0.6 | 1.8 | 50 | 3 | 600 | 20 |
| Papadopoulos [52] | 820 | 50 | 3 | 3 | 714 | 1 | 60 | 6 |
| Stergioulas [48] | 904 | 40 | 1.2 | 7.2 | 80 | 6 | 180 | 12 |
| Vasseljen [58] | 904 | 1.5 | 0.9 | 3.6 | 6 | 4 | 600 | 8 |

Table 5. Characteristics of LLLT regimens with direct tendon application and their treatment variables. Trials given by name of first author, and laser wavelength (nanometer), laser mean output (mW), energy dose per point (Joules), total energy dose per session (Joules), power density (mW/ cm^2^), number of points irradiated, total irradiation time (seconds), number of therapy sessions. (*= estimation made by reviewers).

Table 6

| **Study by first author** | **Laser wavelength (nm)** | **Laser mean output (mW)** | **Energy dose per session (J)** | **Power density (mW/ cm^2^)** | **Points irradiated** | **Total irradiation time (s)** | **No. therapy sessions** |
| --- | --- | --- | --- | --- | --- | --- | --- |
| Haker [46] | 904 | 8 | 1.20 | 16 | 5 | 150 | 10 |
| Lundeberg [47] | 632/904 | 1.56/ 0.07 | 0.09 / 0.004 | 10 | 11 | 60 | 10 |

Table 6. Characteristics of LLLT regimens with acupuncture points application and their treatment variables. Trials given by name of first author, laser wavelength (nanometer), laser mean output (mW), energy dose in per session (Joules), power density (mW/cm^2^) points irradiated total irradiation time (seconds) number of therapy sessions, dichotomized trial result. Acupuncture points used by Haker [46]: Li 10, 11, 12, Lu 5, SJ5. Lundeberg [47] used the same acupuncture points plus the following points: SJ 10, Si 4, 8, H 3, 4, P 3.
